# Promoting Emotional and Social Well-Being and a Sense of Belonging in Adolescents through Participation in Volunteering

**DOI:** 10.3390/healthcare9030359

**Published:** 2021-03-22

**Authors:** Mónica Luque-Suárez, María del Carmen Olmos-Gómez, María Castán-García, Raquel Portillo-Sánchez

**Affiliations:** 1 Department of Sociology, Faculty of Education and Sport Science, University of Granada, 52005 Melilla, Spain; mlsuarez@ugr.es (M.L.-S.); raquel92@correo.ugr.es (R.P.-S.); 2 Department of Research Methods and Diagnosis in Education, Faculty of Education and Sport Science, University of Granada, 52005 Melilla, Spain; mcastan1993@correo.ugr.es

**Keywords:** volunteering, adolescents, emotional well-being, emotional health, sense of belonging

## Abstract

The aim of this study was to analyze the differences within education-related degrees with respect to participation in volunteering. Volunteering motivation promotes and encourages emotional and social well-being and a sense of belonging in university students. This study was based on a total sample of 985 students undertaking Degrees in Early Childhood Education, Primary Education, and Social Education who attended higher education institutions in Northern Africa (Spain) and Eastern Spain. Once the quality parameters of the instrument were determined, the reliability was confirmed, and data collection was initiated. In order to analyze the results, a multilevel study (ANOVA) was conducted by interacting the variables for degrees with three levels (PE = Primary Education; EC = Early Childhood Education; SE = Social Education) and the variable “volunteering is my motivation to feel better”, with five levels (strongly disagree, disagree, unsure, agree, and strongly agree). From the data obtained, it was concluded that there were significant differences between the different degree paths, the assessment covering sociodemographic variables and areas of interest showing that volunteering benefits university students both socially and in their decision-making processes.

## 1. Introduction

Today, universities seek to establish collaborative education networks, in which the university sector works together with companies and community organizations to improve and provide quality education capable of meeting the challenges of and seizing the opportunities provided by social change [1,2,3]. Specifically, university students studying teaching and education believe that experience as a volunteer reinforces the training of these students and their future professional skills [4,5,6]. The literature shows [7,8] that social education degree students have more positive perceptions of volunteering than teaching students due to the social and personal characteristics that define these students, in which empathy, vocational components, and resilience are key elements. Volunteering is now in high demand in professional communities as it provides the experience needed to solve problems in certain systems [9]. The development of voluntary activities may lead to today’s companies wanting motivated young workers who are interested in the progress of their business when they are recruited; that is, these companies would look for young people who can contribute positive ideas, developing their personality at the same time as their labor interests [10,11,12].

Thus, learning programs linked to volunteering are an important aspect to consider, since they equip students with a set of professional skills that improve the quality of their learning outcomes, resulting from the experience of providing a service to a community that enhances the lives of the people within it [13,14].

Students’ education should not be based solely on significant learning, since it is important to consider that good learning takes place through a process of intense reflection that meets all challenges. In turn, these students later help professors experience transformative learning through the students themselves. This concept is based on the idea of learning through participation [15], which is carried out when students undertake activities outside the classroom. Thanks to this new learning experience, students can apply their knowledge and participate in a setting other than the educational institution [16].

Critical reflection on practical experiences enables the activation of transformative learning, since reflection leads students to re-evaluate the problem and their position in order to be able to act [17]. Therefore, it is necessary to clearly understand that to provide an adequate response to the problems that may arise, learning must be based, according to Rodríguez [18], in community outreach activities, while facilitating the theory–practice relationship of a subject and the development of professional competencies, transforming these into a teaching strategy and thereby increasing university education (i.e., student learning occurs within the context of the real needs of the environment and by trying to improve it) [19]. If students do this correctly, it is because their transformative learning has been achieved through reflection and dialogue [20], in contrast to what Freire [21] calls banking education; that is, passive students using their own training process that is disconnected from the environment. For this reason, this link is emphasized, whereby at the same time that theoretical knowledge is acquired, it can be put into practice in different settings [22,23,24].

It has been proven that for the theory–practice connection to be strengthened, experiences where inclusive education skills are reinforced must take place. This is all due to the fact that when students engage in activities related to community service, they learn about situations of inequality and become aware of the stereotypes that they may hold and should overcome [25]. There is scientific research showing that university students who volunteer in schools gain relevant professional experience [26,27,28]. This relationship shows both the improvement of students’ learning and the improvement of the learning and values of university volunteers [29]. Therefore, it must be made clear that the experience of volunteering is totally related to the development of students’ learning and the improvement of their skills as future professionals. Some studies highlight the strong impact of volunteering in disadvantaged contexts where there are very problematic situations that allow students to enhance their experiences [26,30,31].

Many countries are developing programs in educational institutions in which students can participate and acquire all kinds of knowledge from the outside world. This will benefit them, since it adds “glamor” to their curriculum [32]. This acquired knowledge is based on motivations that contribute to understanding the process of the formation and change in an individual’s attitude [33], and in turn is formed on the basis of behavioral beliefs, whereby motivations are the most critical causes of attitudes [34,35]. When working in societies where collectivism is valued, volunteers prioritize group goals [36], generating a high degree of personal satisfaction and commitment [37,38]. In contrast, working in individualistic contexts where selfishness is predominant is related to external rewards associated with materialistic and social motives, such as social networks, skills development, career prospects, and recognition [39,40]. Hustinx and Lammertyn [41,42] called this reflexive volunteering, i.e., when volunteering ceases to be collective and becomes more reflexive, individualistic, or self-centered. Today, volunteers’ motivations include professional development, personal growth, work experience, skill development, and getting a job more easily. Some studies show that young people’s motivations for volunteering are complex and subject to change over time [43,44,45].

Therefore, an educational program in which students can question their intentions in relation to these volunteer activities and make them clear is absolutely necessary. Although the international mobility of volunteers may lead to cases that are detrimental to the image of the volunteer, they may also have many benefits. When a student travels abroad on volunteer missions, once completed they apply that knowledge to their own context from a more critical perspective [46].

Considering the problems that may arise, it is necessary for student volunteers to be able to foresee the consequences of their participation, to be aware of the vulnerability of the group with which they are working, and to be really prepared [47]. These activities are essential for the advancement of social well-being, as they improve the behavior and the quality of life of a society [48]. Therefore, we conducted a questionnaire to identify the influences on university students’ choices for volunteering, based on previous research regarding grade-dependent variables and volunteering motivation. Among the studies on motivation for volunteering, according to Chacon [49], the different regulations (external, introjected, identified, and integrated) that determine whether motivation is intrinsic, extrinsic, or amotivational stand out, which are centered on the reasons for volunteering. Shye [50] also included altruistic facets among student motivations, along with selfish options, whereby the main reason is to try to improve job prospects. Butt et al. [51] linked motivations in four areas of affiliation using an ABCE model, encompassing affiliation (A), beliefs (B), professional development (C), and selfishness (E). Geiser et al. [52] divided motives into intrinsic (interest in a cause, being high in autonomy) and extrinsic (seeking recognition from others), depending on the possibilities of the environment.

As a result of the situations we have experienced and continue to experience, due to COVID-19, all citizens have had to adapt to distance learning. Students must embrace new ways of learning, so individually we must show compassion, respond empathically, and voluntarily help other colleagues who are in difficult situations [53].

In this context, the main objective of this research is to measures the systematic processes that influence students’ volunteering choices and the relationships between volunteer motivation and the degree studied.

## 2. Materials and Methods

### 2.1. Participants

To carry out this study, we analyzed a non-probabilistic (accidental) sample of N = 985 students aged between 17 and 22 years from the first to the fourth year of study at the Faculty of Education and Sport Sciences of Melilla (84.25% female) and the Faculty of Education Sciences of Malaga (74% male) out of those involved in volunteering. Participants represented 93% of the overall number of students (40.4% were first-year students, 29.3% were second-year students, 18.1% were third-year students, and 12.2% were fourth-year students). The students were enrolled in the Degree in Early Childhood Education (21.1%), Degree in Primary Education (43.2%), Degree in Social Education (26.6%), and the Joint Degree in Primary Education, Physical Activity, and Sports Science (primary education specialty) (9.1%).

### 2.2. Instrument

Members of a multidisciplinary team from a department of the Faculty of Education and Sport Sciences of Melilla, which is affiliated with the University of Granada and devoted to the personal, professional, and academic guidance of pre-university and university students, participated in this study. They developed and content-validated a questionnaire to measure the way in which this service influences the choice to participate or not in volunteering, as well as the type of volunteering they do. The questionnaire incorporated sociodemographic variables on the one hand, and on the other hand incorporated variables related to motivation, areas of interest, and volunteering benefits. The instrument design was based on the main theoretical foundations and international recommendations for the development of tests [54]. In order to collect data, this study relied on the voluntary participation of students from the early childhood, primary, and social education degrees and the joint degree taught at the Campus of Melilla and the University of Malaga. Permission was granted to enter university classrooms prior to data collection, in accordance with the Helsinki protocol.

A social survey was taken into consideration in the construction of the questionnaire—the survey on volunteering from the University of Extremadura, the Polytechnic University of Valencia, the Loyola University Andalusia, and the ONGAWA nongovernmental organization. We used the questionnaires shown because in these universities there is a special section for volunteering, such as the University of Extremadura (University Office of Cooperation and Solidarity Action), while the other universities work in areas of social inclusion.

With the purpose of examining the understanding and clarity of the items, the coordination group dealt with the different options, correcting and adjusting the questionnaire according to the corrections made. The final questionnaire consisted of 62 questions divided into two sections, the sociodemographic variables (with six variables), and 56 variables, in order to gather information concerning the motivation to engage in volunteering, the areas of interest, and the perceived benefits of volunteering.

That version was administered to a pilot sample of N = 300 students to assess the content validity and consistency of the questionnaire. The percentage of agreement among the experts was over 90%. Construct validity was established through a semi-confirmatory factor analysis. A Kaiser–Meyer–Olkin index (KMO) value of 0.863 was obtained and a significant value of 0.000 was obtained in Bartlett’s test of sphericity. This permitted us to proceed to factor analysis. The Scree criterion was used, and four factors were obtained. Finally, the results identified the existence of the four factors, explaining 78.072% of the overall variance. The reliability analysis was performed using the Cronbach alpha coefficient, which established that the level of reliability for the questionnaire was between 0 and 1 [55]. With respect to the data, the reliability was 0.932, a figure above 0.5, so it can be said that the reliability was excellent.

### 2.3. Procedure

First, the professors at the Campus of Melilla and the University of Malaga were contacted. The questionnaire was distributed to a sample of N = 985, who participated from a quantitative perspective, through a non-probabilistic, accidental, and causal sampling approach [56]. Both professors and students were informed about the voluntary and anonymous nature of the study, including its aims and objectives. Afterward, the questionnaire was administered in a paper-based format to the students 15 min before the end of a lecture. Data were collected in the first quarter of the course over the period 2020–2021.

### 2.4. Data Analysis

Content validation was performed using the Delphi technique [57] through validation by experts and semi-confirmatory factor analysis. Later, a procedure was used to determine whether the data were parametric or nonparametric, specifically the Levene test [58,59]. The results were parametric, so it was decided to conduct other types of tests using analysis of variance (ANOVA). A multivariate model [60] was used for multiple comparisons, which made it possible to evaluate the groups of participants. Data were analyzed using SPSS 24.0 (IBM SPSS Statistics 24.0 Chicago, IL, USA, 2016).

## 3. Results

In the questionnaire answers (Appendix A.1, template of the questionnaire used), within the context of the reasons why students engaged in volunteering, there were two lines of thought in relation to economic interests. On the one hand, the option “involvement looks good in my curriculum” has the highest mean score among the students of the Degrees in Primary and Early Childhood Education; on the other hand, the option “get my foot in the door to professional employment” had the highest mean score among Social Education students. Regarding the questions related to personal interests—that is, whether “they are encouraged to engage in volunteering by people they are close to”—the highest mean scores were reported by Early Childhood Education students. Finally, when dealing with aspects associated with “solidarity and the desire to help others”, the highest scores were obtained from students within the Degrees in Social Education and Early Childhood Education.

The students who engaged least in volunteering due to economic reasons were those studying the Degree in Early Childhood Education. Primary Education students did not often engage due to the lack of information and interesting activities. The students in the joint degree showed a lack of interest or time.

The areas of greatest interest to Social Education students were those related to socially excluded groups, the environment, human rights, functional diversity, gender and women’s organizations, migration, refugees and asylum seekers, civil protection, and rescue. The students of the Degree in Primary Education show much more concern about other areas, such as biodiversity and wildlife, culture, and public health. Early Childhood Education students were interested in aspects such as childhood and youth, the elderly, culture, and community service. Lastly, the students of the joint degree were much more significantly involved in volunteering in entertainment, leisure, and free time, as well as in sport volunteering.

Finally, Table 1 shows that Social Education students believe, in general, that volunteering can contribute to any aspect, from professional and personal growth to helping others or even finding inner peace and joy.

Table 1 shows the results of the ANOVA conducted on the variables (degree studied and volunteer motivation) and the relationships between them. The multivariate test indicated significant differences and large effect sizes for volunteer motivation (F > 4.256, *p* < 0.001, η2 > 0.253), significant differences regarding the degrees studied (F > 2.294, *p* < 0.001, η2 > 0.298), and significant results for the relationships between volunteer motivation and degree studied (F > 4.178, *p* < 0.001, η2 > 0.297) [61,62,63].

The multivariate test is a data analysis technique that is used to simultaneously analyze the relationships between different levels of the same variable and the relationships between the levels of two different variables [64]. These tests identify covariance effects and allowed us to statistically study the influence of the independent groups (with three levels: PE = Primary Education, EC = Early Childhood Education, SE = Social Education) and the “volunteering is my motivation to feel better” variable (with five levels: strongly disagree, disagree, unsure, agree, strongly agree), using the mean scores at the individual level, with the dependent measures of the systematic processes that influence adolescent students’ volunteering choices.

The results showed large effects on the sample size and the proportion of variance explained (ANOVA) [65] at above 0.14 [66], which is already considered a significant effect, as well as significant differences [67] with respect to volunteer motivation, with more than 25.3% (η2 > 0.253) of the differences being attributed to the notion that volunteering makes them feel better about themselves. Previous studies [66,68,69] have suggested that eta-squared values greater than 14 show a large effect, with r = 10 being a low effect, a medium effect being r = 0.30, and r = 0.50 being a high effect. Likewise, the results obtained showed significant differences with respect to the groups depending on the degree studied. With respect to the variable groups, the sample size (ANOVA) [70] indicated that more than 29.8% (η2 > 0.298) of the differences found were related to the observed changes in the participation in volunteering, the amount, and the type, depending on what was being studied. The values can be attributed to the effect of the “need for belonging” variable. Although that value was higher than 0.14, this represented one-quarter of the surveyed population, which in social terms and based on this study, is highly determinant. Regarding the square sample size for the relationships between the students of the different degrees and volunteer motivation, almost 29.7% (η2 > 0.297) of the differences found were attributed to the effect of “personal improvement” [59,60,61].

The adjustment of the results (Table A1, Appendix A.2) from the ANOVA data revealed a significant association between volunteer motivation and the effect of feeling better about oneself, as well as with the different degrees studied. The results were related to providing help and to considering that volunteering helps them to get a job; looks good on the CV; and promotes their strengths, sense of belonging, and dedication to others, all of which showed significant values (*p* < 0.005); in addition to noting the need to promote volunteering for the elderly, sport, public health, biodiversity, childhood and youth, the environment, and human rights, all of which showed significant values (*p* < 0.005). The importance attached to volunteering was emphasized by the help, fostering of values, life experience, and personal satisfaction variables, all of which showed significant values (*p* < 0.005).

In relation to the degree and volunteering variables, 68% of respondents were motivated by feelings of love of service, 99% by the fact that it is helpful to “get their foot in the door” where they would like to work, and 35.3% by the influence of friends. Furthermore, 25.9% of students volunteered because they were concerned about people who are less fortunate than themselves and 12.41% agreed with the statement “no matter how bad you are feeling, volunteering helps you forget about it”. Once involved, 79.7% of respondents were genuinely concerned about the group they were serving and 7% felt less lonely thanks to volunteering. Likewise, the option “I can make new contacts that might help my business or career” was chosen by 26.51% of respondents; “I can learn more about the cause for which I am working” by 74.4%; “volunteering increases my self-esteem” by 87.8%; “others with whom I am close place a high value on community service” by 10.81%; “volunteering is a good escape from my own troubles” by 88.3%; and “volunteering experience will look good on my CV” by 17.8%. Positive experiences were closely linked to greater intrinsic motivation regarding education and interactions with others for the improvement of social and emotional well-being [71]. Intrinsic motivation was completely related to learning and the achievement of set goals [72].

## 4. Discussion

In this work, we analyzed the relationships between the variables “university degree” and “students’ motivation” with the dependent measures related to participating in volunteering, involving community-based groups at the Faculty of Education and Sport Sciences of Melilla and the Faculty of Education Sciences of Malaga. These data were supported by the study of Costa and McCrae [70], who dealt with the importance of social interactions for the development of kindness, emotional well-being, organization, control, and motivation as a means of ensuring social well-being, while gaining new experiences in the process.

The ANOVA tests showed significant differences and varied effect sizes in one-quarter of the surveyed population and in the relationships between the students of the different degrees and volunteer motivation. Economic status was still a major influential factor of student engagement in volunteering of any kind, as students think that, as a result of their participation, they will receive a payment or that it will help them later, improving their economic situation. It is also worth highlighting personal aspects, considering that volunteering experiences can help them improve and develop themselves, strengthen their self-esteem, and generate new feelings of solidarity and empathy, among others. Special attention was paid to the most vulnerable groups and those who can be helped by participating in these volunteer activities. There was a negative relationship with people with a high level of income and a positive relationship with respect to a person’s working hours [73].

Factor 1, professional development through volunteering, analyzed the action of volunteering from a professional approach. Communities and governments recognize the importance of volunteering to promote sustainable economic development and social inclusion. Participation in volunteering is one of the intermediate indicators for promoting future employment (finding a job) [74,75,76]. Factor 2 focused on personal growth through volunteering. Hustinx [77] discussed the connection between volunteers striving for solidarity and personal development and how this impacts their self-actualization, social contacts, and work experience [78]. For the next factor, economic interest versus solidarity, the altruistic–egoistic duality was analyzed to explain the phenomenon of volunteering. According to this perspective, young people are more likely to engage in volunteering to gain new skills that may lead to new or better employment opportunities [79,80,81]. Finally, factor 4, social skills developed through volunteering, described how the vast majority of studies show that volunteers prefer to see themselves as motivated primarily by “altruistic” reasons, and that these motives score the highest on the value scales, regardless of the volunteer’s age, gender, or background. Understanding is the second highest rated on that scale [82,83,84].

The “university degree variable” came as a great revelation, given that participants were much more interested in volunteering activities from which they could obtain direct learning experiences related to their future career; that is, many were motivated to participate because they knew that these experiences will later improve their curriculum vitae and their position in the job market. Therefore, it is necessary to emphasize the importance of an active role, generating new personal expectations according to the interactions with the environment [85]. This relationship creates a dependency between the student and the environment, or the so-called “learning–service” setting [86]. For example, the students of the joint degree gave the highest scores to sports, although any student should be interested in sporting activities, as it is the best way to create healthy habits. This can be a problem, which together with the characteristics of the city of Melilla, may affect many people due to the fact that many prejudices arise from the idea that teachers are limited to certain topics. Learning must be continuous, and the more open the learning is, the more enriching the experience will be for a person.

## 5. Conclusions

This study aimed to raise awareness and consciousness among the student community about the importance of engaging in volunteering, including the various interests and motivational aspects through which they can be influenced. Volunteering is increasingly being associated with positive traits that help improve mental and physical health and that expand social relationships thanks to effective integration. There are authors who see volunteering as a therapeutic method of coping with feelings of depression or isolation, improving self-esteem and people’s lives by promoting emotional well-being [87,88]. Such situations emerged after the first wave of the coronavirus pandemic due to teleworking, which has modified activities related to face-to-face contact, as well as cognitive aspects, social well-being, and motivation, leading to the restructuring of people’s lives [89].

Economic issues are important for these students, since young Europeans are taking much longer to leave the family home because of their poor economic situation, resulting from a very high unemployment rate, a situation of long-term unemployment, and very poorly paid or short-term jobs [90]. This is their primary reason for volunteering, as many of them are seeking an alternative way to get a job.

Family and friends should encourage you to do what makes you feel professionally fulfilled and what will bring you joy in the future. No one should hinder you or judge whether a certain degree is more male- of female-oriented, since both men and women can practice sports or help people in vulnerable situations. With this study and the knowledge gained from it on the basis of other research studies, we provide new ideas and research directions, although it will never be possible to fully understand volunteering and all the effects that it could encompass. Certain studies have shown that a stereotypical perception of volunteering and volunteers prevails among young people [91,92,93,94].

It is important to note some limitations of the present study. First, the sample could be expanded to more educational fields, which would provide a broader vision of the knowledge about volunteering at an academic level. Second, there is a need for new studies that are able to find evidence of volunteer motivation in relation to the experimental variable of gender, among others. Therefore, we conclude that the results of this study will enable multidimensional analyses in the future.

## Figures and Tables

**Table 1:** Analysis of variance (ANOVA) and effect size (η^2^) results for sums of aggregated scales of promotion of social well-being through participation in volunteering using the volunteer motivation and field of study questionnaire.

Factors		M	SD	CI (95%)		F	*p*	η^2^
				Lower Limit	Higher Limit			
Professional development through volunteering	Careers	3.46	0.891	2.96	4.01	2.391	<0.001	0.323
	Motivation	3.18	0.887	3.01	4.22	4.256	<0.001	0.261
	Careers × motivation	3.82	0.943	3.61	4.27	4.186	<0.001	0.367
Personal growth through volunteering	Careers	3.09	0.767	2.91	3.53	2.498	<0.001	0.298
	Motivation	3.59	0.831	3.24	4.01	4.371	<0.001	0.272
	Careers × motivation	3.52	0.867	3.13	4.12	5.143	<0.001	0.297
Economic interest versus solidarity	Careers	3.71	0.988	3.21	4.21	2.997	<0.001	0.346
	Motivation	3.80	0.963	3.51	4.26	4.715	<0.001	0.253
	Careers × motivation	3.77	1.121	3.29	4.20	7.449	<0.001	0.387
Social skill development through volunteering	Careers	3.19	0.896	3.04	3.51	2.294	<0.001	0.325
	Motivation	3.16	0.795	2.79	3.31	6.261	<0.001	0.266
	Careers × motivation	3.18	0.981	2.93	3.82	4.178	<0.001	0.364

The critical alpha level was adjusted for multiple testing to reduce the type I error (α). Thus, the α-value was divided by the number of pair comparisons for each ANOVA.

## Data Availability

The data presented in this study are available on request from the first author, e-mail: mlsuarez@ugr.es (M.L.-S.).

## Appendix A

### Appendix A.1. Template of the Questionnaire Used: Promoting Social Well-Being through Participation in Volunteering

1.Sex: ___ Female / ___ Male					
2.How would you describe the socio-economic status of your family? ___ High ___ Medium___ Low					
3. Academic year: 1st __ 2nd __ 3rd __ 4th __					
4. Faculty: Education and sport science Melilla (University of Granada) ___/Education Science (University of Málaga) ___					
5.Volunteering is my motivation to feel better.	STRONGLY DISAGREE	DISAGREE	UNSURE	AGREE	STRONGLY AGREE

6. Degree undertaken:

**Degree**
Early Childhood Education
Primary Education
Social Education
Primary Education (Physical Activity and Sports Science)

**What motivated you to become a volunteer?**	**STRONGLY DISAGREE**	**DISAGREE**	**UNSURE**	**AGREE**	**STRONGLY AGREE**
I.1. Service–Love					
I.2. Volunteering can help me to “get my foot in the door” where I would like to work					
I.3. My friends volunteer					
I.4. I am concerned about people who are less fortunate than myself					
I.5. People I am close to want me to volunteer					
I.6. The people I know have a common interest in solidarity					
I.7. No matter how bad I am feeling, volunteering helps me forget about it.					
I.8. I am genuinely concerned about the particular group I am serving					
I.9. By volunteering I feel less lonely					
I.10. I can make new contacts that might help my business or career					
I.11. Doing volunteer work relieves me of some of the guilt over being more fortunate than others.					
I.12. I can learn more about the cause for which I am working					
I.13. Volunteering increases my self-esteem					
I.14. Volunteering allows me to gain a new perspective on things					
I.15. Volunteering allows me to explore different career options					
I.16. I feel compassion toward people in need					
I.17. Others with whom I am close place a high value on community service					
I.18. Volunteering let me learn things through direct hands-on experience					
I.19. I feel it is important to help others					
I.20. Volunteering helps me work through my own personal problems					
I.21. Volunteering will help me to succeed in my chosen profession					
I.22. I can do something for a cause that is important to me					
I.23. Volunteering is an important activity to the people I know best					
I.24. Volunteering is a good escape from my own troubles.					
I.25. I can learn how to deal with a variety of people					
I.26. Volunteering makes me feel needed					
I.27. Volunteering makes me feel better about myself					
I.28. Volunteering experience will look good on my CV					
I.29. Volunteering is a way to make new friends					
I.30. I can explore my own strengths					
**If you have never participated in volunteering activities, please indicate your level of agreement with the following reasons**.	**STRONGLY DISAGREE**	**DISAGREE**	**UNSURE**	**AGREE**	**STRONGLY AGREE**
I.31. Economic reasons					
I.32. Activities are not interesting					
I.33. Lack of information					
I.34. Lack of interest					
I.35. Lack of time					
I.36. Lack of volunteering opportunities in my area					
**What area of volunteering are you most interested in**?	**STRONGLY DISAGREE**	**DISAGREE**	**UNSURE**	**AGREE**	**STRONGLY AGREE**
I.37. Biodiversity and wildlife					
I.38. Excluded groups: Homelessness/Drug abuse					
I.39. Environmental conservation					
I.40. Human rights					
I.41. Functional diversity					
I.42. Childhood and youth					
I.43. Gender and women’s organizations					
I.44. Migration, refugees, and asylum					
I.45. Civil protection and rescue					
I.46. The elderly					
I.47. Entertainment, leisure, and free time					
I.48. Sports					
I.49. Culture					
I.50. Community service					
I.51. Public health					
**What do you think are the benefits of volunteering**?	**STRONGLY DISAGREE**	**DISAGREE**	**UNSURE**	**AGREE**	**STRONGLY AGREE**
I.52.Learning/Professional and academic experience/Better knowledge of reality					
I.53. Personal growth/Development/Enrichment					
I.54. Life experiences					
I.55. New values/Feeling useful in helping others					
I.56. Satisfaction/Inner peace/Joy					

### Appendix A.2. ANOVA and Effect Size (η^2^) Results of the Questionnaire Used: Promoting Social Well-Being through Participation in Volunteering

**Table A1:** ANOVA and effect size (η^2^) results of promoting social well-being through participation in volunteering using the volunteer motivation and field of study questionnaire.

Items	Careers			Volunteer Motivation			Careers × Motivation		
	F	*p*	η^2^	F	*p*	η^2^	F	*p*	η^2^
I.1	0.669	0.459	0.143	0.283	0.623	0.066	0.068	0.808	0.017
I.2	0.975	0.379	0.196	0.412	0.556	0.093	0.099	0.769	0.024
I.3	2.253	0.208	0.360	4.416	0.103	0.525	3.531	0.133	0.469
I.4	0.167	0.703	0.040	0.763	0.432	0.160	2.599	0.182	0.394
I.5	0.303	0.611	0.070	0.167	0.704	0.040	5.530	0.078	0.580
I.6	1.344	0.311	0.252	0.098	0.770	0.024	3.724	0.126	0.482
I.7	4.033	0.115	0.502	0.225	0.660	0.053	1.241	0.328	0.237
I.8	6.000	0.070	0.600	0.828	0.414	0.171	7.972	0.048	0.666
I.9	0.768	0.430	0.161	0.463	0.533	0.104	0.007	0.939	0.002
I.10	4.507	0.101	0.530	6.183	0.068	0.607	2.510	0.007	0.869
I.11	3.227	0.147	0.446	1.946	0.236	0.327	0.028	0.876	0.007
I.12	7.200	0.055	0.643	1.352	0.310	0.253	7.448	0.052	0.651
I.13	9.095	0.039	0.695	11.290	0.028	0.738	0.878	0.402	0.180
I.14	3.044	0.005	0.883	2.967	0.160	0.426	2.322	0.008	0.859
I.15	1.000	0.034	0.714	2.069	0.009	0.847	1.598	0.015	0.806
I.16	0.702	0.449	0.149	0.387	0.568	0.088	1.227	0.330	0.235
I.17	0.138	0.729	0.033	0.076	0.796	0.019	1.081	0.357	0.213
I.18	1.778	0.014	0.816	0.613	0.477	0.133	2.276	0.011	0.835
I.19	0.086	0.784	0.021	0.579	0.489	0.127	3.192	0.149	0.444
I.20	4.320	0.106	0.519	2.797	0.170	0.412	0.883	0.401	0.181
I.21	7.131	0.056	0.641	1.441	0.296	0.265	3.724	0.126	0.482
I.22	3.872	0.120	0.492	0.011	0.922	0.003	0.414	0.555	0.094
I.23	3.000	0.158	0.429	0.414	0.555	0.094	1.986	0.232	0.332
I.24	0.768	0.430	0.161	0.463	0.533	0.104	0.007	0.939	0.002
I.25	1.506	0.287	0.274	0.052	0.831	0.013	1.972	0.233	0.330
I.26	1.920	0.238	0.324	0.149	0.719	0.036	3.531	0.133	0.469
I.27	0.860	0.406	0.177	0.475	0.529	0.106	0.531	0.506	0.117
I.28	0.102	0.766	0.025	0.056	0.824	0.014	0.178	0.695	0.043
I.29	0.218	0.665	0.052	0.008	0.935	0.002	1.229	0.330	0.235
I.30	0.127	0.739	0.031	1.503	0.287	0.273	0.101	0.767	0.025
I.31	3.600	0.131	0.474	10.814	0.030	0.730	2.253	0.208	0.360
I.32	0.533	0.506	0.118	2.225	0.210	0.357	1.241	0.328	0.237
I.33	3000	0.158	0.429	0.414	0.555	0.094	1.986	0.232	0.332
I.34	0.240	0.650	0.057	1.622	0.272	0.289	3.338	0.142	0.455
I.35	0.102	0.766	0.025	1.199	0.335	0.231	0.795	0.423	0.166
I.36	1.202	0.335	0.231	0.663	0.461	0.142	0.002	0.968	0.000
I.37	2.034	0.227	0.337	0.281	0.624	0.066	0.058	0.821	0.014
I.38	1.067	0.360	0.211	0.451	0.539	0.101	2.483	0.190	0.383
I.39	1.375	0.306	0.256	0.759	0.433	0.159	0.181	0.692	0.043
I.40	2.700	0.176	0.403	0.662	0.461	0.142	0.138	0.729	0.033
I.41	0.427	0.549	0.096	5.035	0.088	0.557	3.338	0.142	0.455
I.42	1.796	0.251	0.310	7.496	0.052	0.652	0.181	0.692	0.043
I.43	8.112	0.046	0.670	0.062	0.816	0.015	0.878	0.402	0.180
I.44	6.123	.068	0.320	0.087	0.995	0.087	0.987	0.563	0.158
I.45	2.376	0.198	0.373	1.712	0.261	0.300	2.821	0.168	0.414
I.46	0.656	0.463	0.141	0.001	0.972	0.000	0.265	0.634	0.062
I.47	0.747	0.436	0.157	2.020	0.228	0.336	0.319	0.602	0.074
I.48	0.164	0.706	0.039	0.748	0.436	0.158	0.011	0.923	0.003
I.49	1.796	0.251	0.310	7.496	0.052	0.652	0.181	0.692	0.043
I.50	8.067	0.047	0.669	25.830	0.007	0.866	1.103	0.353	0.216
I.51	0.038	0.855	0.009	2.208	0.211	0.356	0.039	0.852	0.010
I.52	0.253	0.642	0.059	2.230	0.210	0.358	3.201	0.148	0.445
I.53	0.253	0.642	0.059	2.230	0.210	0.358	3.201	0.148	0.445
I.54	1.796	0.251	0.310	0.759	0.433	0.159	0.181	0.692	0.043
I.55	3.227	0.147	0.446	1.946	0.236	0.327	0.028	0.876	0.007
I.56	5.503	0.037	0.704	6.848	0.059	0.631	0.013	0.916	0.003

Note: Multilevel linear adjustment was used to reduce the type I error (α). Thus, the α-value was divided by the number of pair comparisons for each ANOVA.
